# Getting “Inside” Type I IFNs: Type I IFNs in Intracellular Bacterial Infections

**DOI:** 10.1155/2017/9361802

**Published:** 2017-04-26

**Authors:** Deann T. Snyder, Jodi F. Hedges, Mark A. Jutila

**Affiliations:** Department of Microbiology and Immunology, Montana State University, Bozeman, MT, USA

## Abstract

Type I interferons represent a unique and complex group of cytokines, serving many purposes during innate and adaptive immunity. Discovered in the context of viral infections, type I IFNs are now known to have myriad effects in infectious and autoimmune disease settings. Type I IFN signaling during bacterial infections is dependent on many factors including whether the infecting bacterium is intracellular or extracellular, as different signaling pathways are activated. As such, the repercussions of type I IFN induction can positively or negatively impact the disease outcome. This review focuses on type I IFN induction and downstream consequences during infection with the following intracellular bacteria: *Chlamydia trachomatis*, *Listeria monocytogenes*, *Mycobacterium tuberculosis*, *Salmonella enterica* serovar Typhimurium, *Francisella tularensis*, *Brucella abortus*, *Legionella pneumophila*, and *Coxiella burnetii*. Intracellular bacterial infections are unique because the bacteria must avoid, circumvent, and even co-opt microbial “sensing” mechanisms in order to reside and replicate within a host cell. Furthermore, life inside a host cell makes intracellular bacteria more difficult to target with antibiotics. Because type I IFNs are important immune effectors, modulating this pathway may improve disease outcomes. But first, it is critical to understand the context-dependent effects of the type I IFN pathway in intracellular bacterial infections.

## 1. Introduction

Originally discovered for their antiviral activity, type I interferons (IFNs) are now known to also impact a variety of infectious and inflammatory disease states that are not exclusive to the antiviral response [1]. In fact, the story of type I IFNs reaches beyond the protective role for which they were discovered. Not only can these potent cytokines defend the host from viral infection but they can also promote persistent viral infection in some settings [2–5]. Similarly, these cytokines can either harm or benefit the host in autoimmune diseases [6–9]. Furthermore, type I IFNs are treatments for some viral infections and autoimmune diseases, stressing the importance of understanding their impact on the host immune system [3, 10]. Not surprisingly, type I IFNs can have opposing effects during both intracellular and extracellular bacterial infections as well. Because type I IFNs are produced during an immense number of distinct infections and inflammatory diseases, their importance from an evolutionary and immune standpoint is clear.

Type I IFNs are one of three types of interferons. Type I, II, and III IFNs are classified based on activity, structure, and corresponding receptor type. There are many groups of type I IFNs: IFN-*α*, *β*, *κ*, *ω*, *τ*, and *ε* [11]. Of the type I IFNs, IFN-*β* and IFN-*α* are most well-studied. There is only one IFN-*β* while there are 12 subtypes of IFN-*α* in humans and 14 in mice [1]. Type II IFN consists of only IFN-*γ* while there are four types of type III IFNs in humans and two in mice [12]. Interferons can induce immune changes at minimal concentration; thus, tight regulation of interferon responses is required and may be determined, in part, by interferon receptor distribution and expression [13, 14]. Most, if not all, cells can respond to and produce type I IFNs, but plasmacytoid dendritic cells (pDCs) are some of the most robust producers. They can generate 10-fold more IFN-*α* than monocytes [15]. Although pDCs will not be discussed in relation to the intracellular bacteria covered in this review, their role as robust type I IFN producers is likely an important part of the immune response.

Despite disparate survival strategies, intracellular and extracellular bacteria are both capable of inducing type I IFNs with some overlap in induction pathways. Whereas intracellular bacteria can activate intracellular sensors from within the phagolysosome or cytoplasm, extracellular bacteria introduce type I IFN-stimulating ligands into the cytosol via pore-forming proteins or other means [16–18]. This review concentrates on the actions of type I IFNs in the context of intracellular bacterial infections. The functions of these cytokines in other microbial infections, cancer, and autoimmunity have been extensively reviewed elsewhere [8, 19–21]. The following sections will describe pathways leading to, and the downstream results of, type I IFN production.

## 2. Induction of Type I IFNs

Pathogen-associated molecular patterns (PAMPs) are sensed by their cognate pattern recognition receptor (PRRs), which leads to transcription of many gene products, including type I IFNs. Toll-like receptors (TLRs), C-type lectin receptors (CLRs), retinoic acid-inducing gene I- (RIG-I-) like receptors (RLRs), and nucleotide-binding oligomerization domain- (NOD-) like receptors (NLRs) are all PRRs. TLRs and CLRs are transmembrane receptors, whereas RLRs and NLRs reside in the cytoplasm [22]. Multiple PRRs can be engaged during infection, leading to an orchestrated innate immune response that is specific to a pathogen's repertoire of PAMPs.

TLRs sense PAMPs, including nucleic acids and lipoproteins from invading pathogens, and either are expressed on the cell surface or are present in endosomes and lysosomes in immune cells. Endosomal TLRs recognize viral and bacterial nucleic acids and lead to type I IFN production; these are TLR3, 7, and 9 in mice and humans [23]. While TLR3 is mostly expressed intracellularly, cell surface TLR3 has been observed on human dendritic cells, macrophages, endothelial cells, and synovial fibroblasts of rheumatoid arthritis patients [24]. TLR4, found on the cell surface, recognizes the gram-negative bacterial component, lipopolysaccharide (LPS). Following TLR-ligand binding, signaling occurs via two main adaptor protein pathways, myeloid differentiation factor 88 (MyD88) or TIR domain-containing adaptor-inducing IFN-*β* (TRIF) [22]. All TLRs signal through MyD88 except TLR3, which utilizes only TRIF, and TLR4 which utilizes TRIF or MyD88 [23, 25]. TRIF signaling leads to type I IFN production via TANK-binding kinase- (TBK-1-) mediated activation of transcription factors, interferon regulatory factors (IRF) 3 or 7. Alternatively, downstream of MyD88, inflammatory genes are induced via transcription factor, nuclear factor kappa-light-chain-enhancer of activated B cells (NF-*κ*B) [26]. However, endosomal MyD88 signaling leads to type I IFN production via IL-1R1-associated protein kinase 4- (IRAK4-) mediated activation of IRF7 [27].

Of the nucleic acid sensing TLRs, TLR9 is the only DNA sensor and is expressed on endosomes or cell surfaces [28–30]. TLR9 specifically detects unmethylated CpG DNA from bacteria [31]. TLR9 has been shown to be important during infection with *S. enterica* serovar Typhimurium (SesT), *L. pneumophila*, and controversially in the context of *B. abortus* [32–36]. There is little or no direct evidence of type I IFN production downstream of TLR9 in *L. pneumophila* and SesT infection and disparate results regarding *B. abortus*; thus, it will not be discussed in detail [32–34]. Several DNA sensors, mostly cytoplasmic, have been identified. They can lead to type I IFN production, as well as inflammasome formation, autophagy, necrosis, apoptosis, and production of inflammatory mediators [37]. Cytosolic DNA sensors can be categorized into two distinct signaling pathways, namely, the absent in melanoma 2 (AIM2)/casapase-1 inflammasome pathway, which activates pro-IL-1*β* and pro-IL-18, and the interferon stimulatory DNA pathway (ISD), which leads to type I IFN production [38]. The main ISD pathway involves cyclic GMP-AMP (cGAMP) synthase (cGAS) and stimulator of IFN genes (STING). Upon viral or bacterial DNA sensing, cGAS generates cyclic dinucleotides (CDNs), like cGAMP, which activate STING, an endoplasmic reticulum-associated protein that induces production of type I IFNs in a TBK1/IRF3-dependent manner [39]. This mode of DNA detection has been observed in *M. tuberculosis*, *L. monocytogenes*, and *C. trachomatis* infection [40–44]. Finally, type I IFN production also occurs following DNA or polyI(dA:dT) sensing via RNA polymerase III (Pol III), which converts DNA into RNA ligands for RIG-I [45–47]. There has been little progress in understanding the mechanism of Pol III-mediated type I IFN production and blocking Pol III has no effect on type I IFN transcript levels in certain cell types [48]. Nonetheless, this pathway has been implicated in DNA detection and subsequent type I IFN production during infection with *L. monocytogenes* and *L. pneumophila* [47, 49]. Though there is still more to learn in regard to detection of DNA and type I IFN induction, it is clear that DNA sensing is integral to innate immune recognition of intracellular pathogens.

Type I IFNs are produced in response to RIG-I-like receptor (RLR) sensing of dsRNA, mostly derived from viruses. However, some studies reveal evidence of RLR involvement during intracellular bacterial infections with *L. monocytogenes*, SesT, and *L. pneumophila* [50]. RIG-I, melanoma differentiation-associated gene 5 (MDA5), and laboratory of genetics and physiology 2 (LGP2) are all RLRs. RIG-I recognizes short viral dsRNA or ssRNA with a 5′-triphosphate group while MDA5 recognizes long dsRNA and its synthetic analog, polyI:C [51, 52]. After ligand recognition, RIG-I and MDA5 caspase activation and recruitment domains (CARDs) interact with a mitochondrial/peroxisomal-associated protein, interferon promoter stimulator 1 (IPS-1, also called MAVS, Cardif or VISA). This is followed by phosphorylation of NF-*κ*B or IRF-3, similar to processes that occur following TLR stimulation [53]. MDA5 and RIG-I sequences are conserved in the C terminus and helicase domain of LGP2 but unlike the other RLRs, LGP2 does not contain a CARD region [54]. This feature has made it challenging to elucidate the role of LGP2. Type I IFN induction via these receptors is becoming an important pathway to understand in the context of intracellular bacterial infections.

Like RLRs and DNA sensors, NLRs are cytosolic sentinels of pathogen invasion. NLRs are expressed in both immune and nonimmune cells, such as epithelial cells, and thus far, there are 22 recognized NLRs in humans and 34 in mice [55]. Similar to TLRs, PAMPs bind NLRs, leading to activation of inflammatory pathways via mitogen-activated protein kinase (MAPKs) and NF-*κ*B, inflammasome activation, and type I IFN production [56]. For example, NF-*κ*B activation can occur via NOD1 or NOD2 in response to peptidoglycan from gram-negative or gram-positive bacteria, respectively [57, 58]. Following ligation, NLR proteins oligomerize and interact via their CARD domains with adaptor protein, receptor interacting protein kinase 2 (RIP2 or RIPK2), which is required for both NF-*κ*B and MAPK pathway activation and inflammatory gene expression [55, 56]. In addition to activation of proinflammatory cascades which have been reviewed elsewhere [55, 56, 59], NLRs have been implicated in induction of type I IFN expression via IRF7 or 3 activation [60, 61]. Given the large number of NLRs expressed across species, it is evident they are important innate intracellular sensors.

C-type lectin receptors (CLRs) are transmembrane innate receptors that are known for their roles in antifungal immunity but more recently have been shown to play a role in innate immunity to bacteria, viruses, and helminths [62, 63]. Carbohydrate recognition domains on CLRs allow these receptors to interact with not only various carbohydrate motifs but lipids and proteins that may also be present on pathogenic microbes. CLRs can be divided into three categories: immunoreceptor tyrosine-based activation motif domains (ITAM) and spleen tyrosine kinase (Syk), Syk-independent immunoreceptor tyrosine-based inhibition motif domains (ITIM), and those which do not clearly signal through either ITAM or ITIM. A number of bacteria, including the intracellular bacteria *M. tuberculosis*, are known to signal through CLRs like DC-SIGN and Dectin-1. But, thus far, CLR-induced suppression or induction of type I IFNs has not been documented in the context of intracellular bacterial infections [63].

## 3. Type I IFN Signaling

Once type I IFNs are produced and released, they can interact with the same cell that produced them in an autocrine manner or bind to other cells in a paracrine fashion. All type I IFNs signal through a common receptor composed of two chains, IFNAR1 and IFNAR2 [64]. IFN-*β* can also signal through IFNAR1 alone to induce an entirely unique subset of genes, suggesting that abundance of each, IFNAR1 and IFNAR2, may influence downstream gene expression [13, 65]. The conventional and most well-studied pathway of type I IFN signal transduction is the JAK-STAT pathway, but non-STAT signaling pathways exist as well. Non-STAT pathways include the MAPK pathways, mTORC2 pathways that are dependent on Akt, and the protein kinase C (PKC) pathway [66].

Following IFNAR receptor engagement, JAK1 and TYK2 phosphorylate STAT1 and STAT2, which then dimerize and form a complex with IRF9. This complex, called interferon-stimulated gene factor 3 (ISGF3), translocates to the nucleus where it binds interferon-stimulated response elements (ISREs) leading to expression of interferon stimulated genes (ISGs) [67]. In addition to STAT1 and STAT2 heterodimers, other STAT homo- or heterodimers are induced by both type I and type II IFNs. These can bind IFN-*γ*-activated sites (GAS), which leads to production of yet another unique set of genes [66, 68].

Clearly there are numerous pathways that lead to induction of type I IFNs and cellular recognition of type I IFNs can lead to differing outcomes. In most cases involving innate responses to viral infection, type I IFNs are beneficial. In contrast, when considering intracellular bacterial infection, the roles of type I IFNs are less defined and clearly need to be considered individually. In the following sections, we will discuss bacteria that reside and replicate inside cells and the role that type I IFNs play in their pathogenesis.

## 4. Type I IFN Production during Infection with Intracellular Bacteria

### 4.1. *Chlamydia trachomatis* and *C. muridarum*

Some of the first evidence for type I IFNs as antibacterial effectors was observed in elegant experiments with *C. trachomatis* in the early 1970s. Mice treated with Newcastle disease virus or polyI:C and challenged with aerosolized *C. trachomatis* had postponed mortality [69]. However, these results were dependent on the route of virus or polyI:C administration. Other early experiments showed that treating human or mouse cell lines with type I IFN before infection with *C. trachomatis* decreased infectivity of the pathogen [70, 71]. A possible explanation for these antibacterial effects is the ability of type I IFNs to effect important factors for bacterial growth. Type I IFNs can deplete intracellular iron and effect L-tryptophan catabolism via indoleamine 2,3-dioxygenase (IDO) [71, 72]. In addition, when IFN-*α* is combined with IFN-*γ* or TNF-*α* in vitro, the antichlamydial effect of each cytokine alone increases [71]. This suggests that, in vivo, these cytokines may act together to control infection. However, these results are not replicable in the genital infection model using *C. muridarum*. In these experiments, mice lacking the type I IFN receptor (IFNAR−/−) have reduced bacterial burden and clear the infection more quickly than wild-type mice [73]. The decrease in bacterial burden in IFNAR−/− mice is attributed to increased chlamydial-specific CD4 T cell responses and increased CXCL9, a cytokine responsible for T cell recruitment [73]. Finally, recent in vitro infection models suggest that the DNA sensor, cGAS, detects chlamydial DNA which leads to expression of IFN-*β* via STING [44]. Although initial studies suggested that type I IFNs were important for host survival during *C. trachomatis* infection, utilizing a different model and strain-provided opposite results. Thus, it is not clear whether type I IFNs benefit or harm the host in this context.

### 4.2. *Listeria monocytogenes*

Type I IFNs are induced during infection with gram-positive *L. monocytogenes*, which is engulfed by macrophages/monocytes. Rather than being destroyed, the organism survives and replicates within these cells. Survival is due to production of lysteriolysin O, a haemolysin that causes rupture of the phagosome and translocation of *L. monocytogenes* into the cytoplasm where it can be detected by multiple PRRs [67, 74]. The earliest evidence documenting the relationship between *L. monocytogenes* and type I IFN production was observed in 1967 when a “viral inhibitor” was measured in the blood of *L. monocytogenes*-infected chickens [75]. Follow-up work confirmed the production of type I IFNs during *L. monocytogenes* infection [76, 77].

Immune responses to *L. monocytogenes* occur via both TLR-dependent and TLR-independent signaling pathways, within phagosomes and in the cytosol, respectively [78]. These pathways involve STING, RIG-I, or muramyl dipeptide (MDP) sensor, NOD2, in conjunction with TLR signaling [79–81]. Both *L. monocytogenes*-derived DNA and CDNs induce production of type I IFNs [82–84]. Hansen et al. revealed that DNA is a stronger inducer of IFN-*β* in human compared to mouse cells, in which CDNs are more potent stimulators of IFN-*β* [43, 84, 85]. In mouse cells, bacterial DNA is detected via IFI16, cGAS, and STING [43]. Although, it should be noted that recent work has shown IFI16 and other AIM2-like receptors (ALRs) are dispensable for detection of intracellular DNA and subsequent type I IFN production [38]. Thus, the IFI16-cGAS-STING axis described by Hansen et al. should be reexamined.

Type I IFNs are also produced downstream of RIG-I during infection with *L. monocytogenes*. *L. monocytogenes*-secreted RNA, but not RNA obtained from a *L. monocytogenes* lysate, induces IFN-*β* production via RIG-I and MDA5 [49]. This suggests that live *L. monocytogenes* is required for type I IFN induction and it may be secreting RNA into the cytoplasm. Furthermore, secreted DNA from *L. monocytogenes* is transcribed into RNA by Pol III, inducing IFN-*β* via RIG-I [49]. RIG-I and CARD9 are also involved in detection of RNA, inflammasome activation, and IL-1*β* production. These signaling events are dependent on the bacterial secretion system, SecA2, which may release nucleic acids into the cytosol where they can be detected [49]. While human macrophage cell lines can produce type I IFNs in response to both *L. monocytogenes*-derived DNA and RNA, nonimmune cells like human hepatocarcinoma and colon carcinoma cells respond to bacterial RNA in a RIG-I-dependent manner but cannot respond to DNA [80]. Understanding type I IFN production in both nonimmune cells and monocytic cells independently is important because *L. monocytogenes* encounters and infects both cell types.

In addition to nucleic acid-mediated bacterial detection and type I IFN production, NOD2 recognizes cell wall components of *L. monocytogenes* and together with TLR signaling, induces IFN-*β* [81]. Type I IFN responses are most robust when both DNA detection and NOD2 detection of MDP occur simultaneously [81]. Leber et al. predicted that the cytosolic response to DNA may occur via DNA-dependent activator of IFN-regulatory factors (DAI). However, the role of DAI in this setting is no longer supported [86]. More recent studies suggest that STING or RIG-I, via Pol III, act as nucleic acid-sensing pathways for *L. monocytogenes*-induced type I IFN production [43, 49, 80]. The variety of mechanisms of detection for *L. monocytogenes* PAMPs and subsequent induction of type I IFNs indicate an important role for these cytokines.

Initial studies suggest that type I IFN production is harmful to the host in *L. monocytogenes* infection. This is evidenced by resistance of IFNAR- or IRF3-deficient mice to infection and more severe infection upon type I IFN induction via polyI:C treatment [87]. Additionally, STING-dependent type I IFN production correlates with *L. monocytogenes* pathology and prevents cell-mediated immunity because STING- and IRF3-deficient mice display enhanced cell-mediated immunity [79]. Resistance in IFNAR−/− mice is due to decreased lymphocyte and macrophage apoptosis because type I IFNs induce production of proapoptotic genes like *Daxx* and *Trail* [87, 88]. Furthermore, increased peripheral IL-12p70 and production of TNF-*α* by CD11b+ macrophages is observed in IFNAR-deficient mice [89]. This leads to more productive bacterial clearance [87–90]. Additional mechanisms of type I IFN-mediated morbidity and mortality include cross talk between type I IFNs and IFN-*γ*, increased cell-to-cell spread, and decreased splenic neutrophil recruitment [74, 91, 92]. *L. monocytogenes*-infected macrophages secrete type I IFNs which downregulate their own cell surface IFN-*γ* receptor expression [74]. This effect is also noted on dendritic cells during systemic infection [74]. Downregulation of IFN-*γ* receptor occurs due to type I IFN-mediated recruitment of transcriptional regulators, early growth response factor 3 (Egr3), and NGFI-A binding protein 1 (Nab1), to the *ifngr1* promoter and subsequent gene silencing [93]. As a result, myeloid cells are less responsive to IFN-*γ* and more susceptible to infection [74]. Furthermore, *L. monocytogenes*-infected IFNAR−/− mice have increased numbers of IL-17A-secreting *γδ* T cells that attract neutrophils, which aid in bacterial control [91]. Type I IFN signaling also directly affects the intracellular motility of *L. monocytogenes* by impacting bacterial ActA polarization and promoting cell-to-cell spread [92].

Though there are many reports displaying the detrimental effects of type I IFNs during *L. monocytogenes* infection, other investigations suggest that these results depend greatly on route of infection. Whereas most of the previously mentioned studies infect intravenously (i.v.), models utilizing foodborne infection, the natural route of infection, show that IFNAR-deficient mice are not more resistant to infection [94]. Furthermore, type I IFN-induced apoptosis and decreased neutrophil recruitment to the spleen during i.v. infection are not apparent during oral infection, and decreased IFN-*γ* receptor expression on myeloid cells occurs independently of type I IFNs [94]. Finally, wild-type mice infected intraperitoneally (i.p.) have more severe infection compared to wild-type mice infected orally. Upon oral infection, mice lacking type I IFN responses have worse inflammatory pathology of the liver and delayed protective cytokine responses [95]. Thus, the effects of type I IFN signaling may depend on route of infection and target tissue.

### 4.3. *Mycobacterium tuberculosis*

Type I IFN production favors survival and replication of the pathogens *M. tuberculosis* and *M. bovis*. *M. tuberculosis* can infect macrophages and persist in the host for life [96]. The detrimental effect of type I IFNs during this disease has been observed in multiple studies utilizing IFNAR−/− mice which show increased survival and decreased bacterial burden [97–99]. Elevated levels of type I IFNs in humans are associated with greater *M. tuberculosis* infection and pathology, providing further evidence for the role of type I IFNs in maintaining this infection [100]. Unlike *L. monocytogenes*, *M. tuberculosis* does not require escape from the phagosome to trigger type I IFN responses [101]. *M. tuberculosis* expresses a type VII secretion system, called ESX1, which is correlated with type I IFN production. ESX1 is responsible for secretion of *M. tuberculosis* factors, like bacterial DNA, into the cytosol [99, 102].

Type I IFNs are induced downstream of cGAS and STING during infection with *M. tuberculosis* [41, 42]. *M. tuberculosis* infection of macrophages also leads to inflammasome activation and subsequent IL-1*β* secretion. This can occur via NLRP3, which senses potassium ion efflux, or via AIM2 [103, 104]. Wasserman et al. determined that cGAS is required for type I IFN induction in response to *M. tuberculosis* in both mouse and human macrophages. However, rather than direct detection of bacterial CDNs via STING, cGAS must produce CDNs that are detected by STING [41]. Despite compelling evidence for the cGAS/STING axis being a major component of type I IFN production and *Mycobacterium* survival, other investigations have contrasting conclusions. The major finding of these contradictory studies is that cGAS and STING-mediated autophagy is of greater importance to host survival than type I IFN induction [40, 42]. These disparate results concerning type I IFN induction could potentially be explained by differences in in vitro and in vivo studies, unknown variations across institutions, and differences in dose, strain, and timing.

Type I IFNs are also produced during *M. tuberculosis* infection as a result of activation of the NOD2/RIP2/IRF5 axis. Briefly, in vitro studies in mouse bone marrow-derived macrophages (BMDMs) suggest a mechanism of type I IFN production which is dependent on ESX1 expression, detection of *M. tuberculosis*-derived MDP via NOD2 and downstream activation of RIP2, TBK1, and IRF5 [101]. Additionally, RIP2-dependent induction of type I IFNs is 10–100-fold higher when using N-glycolyl MDP from *M. tuberculosis* compared to that when using typical bacterial N-acetylated MDP [101]. Collectively, it is apparent that this organism evolved an efficient means of persisting that is likely dependent on type I IFNs and the cGAS/STING axis or other type I IFN-inducing pathways.

The probacterial effects of type I IFNs have been demonstrated multiple times in the context of *Mycobacterium* species. It is hypothesized that *M. tuberculosis* has evolved to counter the inflammatory effects of IL-1*β* by inducing type I IFNs. For example, treatment with type I IFNs greatly increases susceptibility to *M. tuberculosis* and decreases the ability of macrophages to control *M. bovis* infection [105, 106]. Additionally, IL-10-produced downstream of type I IFNs decreases the antibacterial activity of IFN-*γ* in macrophages, an effect reliant on cGAS and STING [20, 41]. However, another group determined that the negative impact of type I IFNs during disease was due to overt inflammation rather than inhibition of inflammation. Using naturally susceptible 129S2 mice, it was shown that depleting IFNAR1 rescues these mice [98]. In this model, expression of IFNAR1 on both hematopoietic and nonhematopoietic cells increases death of alveolar macrophages, chemokine expression, and neutrophil recruitment to the lung, resulting in fatal inflammation [98]. A recent study suggests that the negative effect of type I IFNs is dependent on IFN-*γ* expression. In the absence of IFN-*γ*, type I IFNs can promote alternative macrophage activation that favors host protection [107]. This may have an impact on human health as some humans have genetic deficiencies in IFN-*γ* signaling. Altogether, the findings suggest that interfering with type I IFN signaling may be a novel therapeutic approach to treatment of *M. tuberculosis* infection.

### 4.4. *Salmonella enterica* serovar Typhimurium

SesT is a gram-negative gastrointestinal bacterium, which can survive in intestinal cell vacuoles and within macrophages [108, 109]. SesT expresses two type III secretion systems (T3SS), *Salmonella* pathogenicity island (SPI-1) and 2 (SPI-2), which are responsible for secretion of effector proteins into host cells and bacterial survival [110]. Infection with SesT induces complex inflammatory cascades, including activation of multiple inflammasome pathways, autophagy, and induction of type I IFNs [111–114]. SesT can induce type I IFNs downstream of TLR4 and TLR3 via TRIF or via RIG-I [34, 112–114].

Mice deficient in inflammasome protein caspase-1 are more susceptible to SesT infection [115, 116]. Follow-up studies show that these caspase-1-deficient mice are naturally deficient in caspase-11 as well, a noncanonical inflammasome caspase [117]. Broz et al. sought to parse out the differential effects of caspase-1 and caspase-11 during infection with SesT and, in doing so, revealed an interesting role for type I IFNs [112]. Type I IFNs produced following TLR4/TRIF stimulation, likely via LPS, are required for caspase-11-induced macrophage death and bacterial release. This is detrimental to the host when caspase-1 is absent as it is necessary for neutrophil-mediated control of SesT released by pyroptotic macrophages [112, 118]. Although early studies show that mice treated with IFN-*α*/*β* are protected from lethality upon intragastric SesT infection, more recent data suggests type I IFNs can benefit or harm the host depending on the functionality of caspase-1 [112, 119]. Furthermore, i.v.-infected IFNAR−/− mice have increased survival due to lack of type I IFN-mediated macrophage necroptosis [120]. Upon type I IFN signaling, IFNAR associates with RIP1 which leads to cell death via RIP1/RIP3 necroptosis. This only occurs when caspase-8 is blocked, which is relevant to SesT infection as caspase-8 decreases during infection [120, 121]. Thus, multiple pathways of SesT-induced cell death that advance pathogenesis are facilitated by type I IFNs.

IFN-*β* treatment is detrimental to the host because it can decrease transcriptional responses in SesT-infected macrophages, specifically IL-18, IL-1*β*, and neutrophil chemokines CXCL1, 2, and 5 [122]. In addition, treatment with IFN-*β* increases SesT-induced IFN-*β* mRNA [122]. The effect of IFN-*β* on macrophage transcriptional responses is dependent on IL-10, but type I IFN-mediated macrophage necroptosis is not [122]. Oral and i.p. infection confirm the harmful effects of IFN-*β* that correlate with decreased IL-1*β* and CXCL2 expression and dampened neutrophil influx to the small intestine [122]. These results suggest that type I IFN, induced by SesT, decreases inflammatory responses thereby allowing for greater SesT propagation.

The mechanisms of type I IFN-mediated harm depend on route of infection but are correlated with macrophage responses or survival, highlighting the importance of this immune cell during SesT infection. However, nonphagocytic cells also play an important role because, in these cells, type I IFNs are induced by RIG-I in response to SesT [114]. Mouse fibroblasts produce IFN-*β* downstream of RIG-I upon infection whereas macrophages produce IFN-*β* downstream of TLR signaling [114]. These results are relevant in vivo given that SesT can also infect nonphagocytic and nonimmune cells. Additionally, it is important to consider the potential of type I IFNs to act on alternative aspects of immunity, for example, the gut microbiota. Some patients who develop respiratory tract infections with influenza also experience gastrointestinal symptoms of unknown etiology [123]. Influenza-induced type I IFNs produced in the lung negatively alter the gut microbiota of mice, causing them to be more susceptible to SesT infection [124]. The two main observations of this study were that influenza-induced type I IFNs created a dysbiotic gut microbiota and dampened inflammatory and antimicrobial responses in the gut, increasing susceptibility to SesT infection. Thus, the impact of type I IFN production may have broader impacts than previously thought.

Even though most recent evidence highlights the negative impact of type I IFNs in SesT infection, type I IFNs can also protect the host during infection with less pathogenic strains [113, 119, 125]. Oral infection with a noninvasive strain of SesT that lacks SPI-2 reduces TRIF-dependent IFN-*β* induction, which leads to cell-mediated IFN-*γ* production and subsequent antimicrobial macrophage activity [113]. This strain of SesT suppresses the antibacterial focal adhesion kinase (FAK)/Akt/mTORC1 autophagy pathway preventing SesT-derived-PAMPs from signaling through TLR3 and TLR4 and subsequent protective IFN-*β* production [113]. However, these results may not be comparable to other investigations that utilize invasive SesT.

### 4.5. *Francisella tularensis* and *F. tularensis* Subspecies *novicida*

The gram-negative bacteria, *F. tularensis* and *F. novicida*, are the etiologic agents of tularemia. *F. tularensis* is more virulent than *F. novicida* but the two are sometimes used interchangeably in experiments. Similar to *L. monocytogenes*, *F. tularensis* is engulfed by macrophages, then escapes the host cell phagosome, allowing cytosolic replication and subsequent localization to autophagosome-like vacuoles [126, 127]. However, it is now known that *F. tularensis* can infect dendritic cells (DCs) and neutrophils in addition to macrophages [128, 129]. *Francisella* pathogenicity island proteins and related transcription factors like MgIA and MgIB are essential for virulence, escape from the phagosome, type I IFN induction, and inflammasome activation which occur within the cytosol [130–133]. Furthermore, *F. tularensis* has a unique LPS, similar to that of *C. burnetii* that aids in evasion of host immune responses [134]. Together, these responses allow *F. tularensis* to survive, replicate, and escape innate host responses.

Early studies discovered that *F. novicida* induces type I IFN production in a manner independent of TLRs, NOD1/2, RIP2, ASC, Ipaf, IPS-1, RIG-I, and MDA5 but dependent on IRF3 [126]. Initially, the cytosolic sensor for *F*. *novicida* was unidentified but it is now known that cGAS and IFI204 cooperatively detect dsDNA derived from *F. novicida*, in turn activating STING which leads to expression of type I IFNs [135]. However, as previously discussed, Gray et al. determined that ALRs, like IFI204, are not involved in type I IFN induction in response to DNA [38]. Thus, further investigation into the involvement of IFI204 in this response to *F. novicida* is required.

Type I IFNs produced via the cGAS-IFI204-STING axis then signal in a paracrine manner to other cells and are necessary for activating the inflammasome, an event that causes macrophage death [126, 136]. In more detail, *F. tularensis*-induced type I IFNs drive expression of transcription factor IRF1, that then causes expression of guanylate binding proteins (GBPs) [137, 138]. GBPs have multiple antimicrobial functions against intracellular pathogens including disruption of pathogen-containing vacuoles [138, 139]. In this case, the GBPs directly disrupt the membrane integrity of cytosolic *F. tularensis* [137, 138]. This allows release of bacterial DNA to the cytosol, activation of the AIM2 inflammasome and subsequent IL-18 and IL-1*β* production and pyroptotic cell death. However, it is still unknown how exactly *F. tularensis* DNA reaches the cytosol to initially signal via cGAS, IFI204, and STING. It is hypothesized that low levels of DNA are required for cGAS detection whereas much greater levels are required for AIM2 activation [136]. These experiments suggest that type I IFNs are necessary for controlling spread of *F. tularensis* by eliminating macrophages which act as replication niches.

Though many in vitro experiments utilizing macrophage cell lines and some in vivo infection models suggest that type I IFN-mediated inflammasome activation is important for bacterial control, other studies prove otherwise. Upon intradermal infection with *F. novicida*, IFNAR−/− mice survive better than wild-type mice due to greater numbers of IL-17A-secreting *γδ* T cells [91]. Similar to *L. monocytogenes*, *γδ* T cell-derived IL-17A is important for splenic neutrophil recruitment and bacterial control. This also occurs during intranasal infection with *F. tularensis*. Therefore, in these in vivo models, type I IFNs are a detriment to the host because they decrease antibacterial activity of *γδ* T cells and neutrophils [91]. It is evident that type I IFNs may have differing effects on the host during tularemia thus their role may depend greatly on bacterial burden and differences between strains and timing during infection.

### 4.6. *Brucella abortus*

*B. abortus* is a gram-negative bacterium that can infect and survive within macrophages and DCs [140, 141]. Survival strategies include subverting phagosome/lysosome fusion creating a *Brucella*-containing vacuole (BCV) in which the bacterium can replicate, induction of DC death, and more virulent strains can inhibit macrophage cell death [140, 142, 143]. The type IV secretion system, virB, is necessary for developing the BCV. The first evidence connecting *B. abortus* with type I IFNs was observed when IFN-*α* was detected in the serum of mice treated with heat-killed *B. abortus*. This effect was diminished in mice lacking TLR9, demonstrating that *B. abortus*-induced type I IFN production was dependent on TLR9 [35]. Furthermore, *B. abortus*-infected DCs secrete significantly less IFN-*β*, among other cytokines, compared to DCs infected with *Salmonella*, and have altered maturation. This suggests that *B. abortus* is altering DC activation and maturation, resulting in dampened cytokine secretion [141].

A later study by de Almeida et al. confirmed the connection between *B. abortus* infection and type I IFN production, elucidated potential mechanisms of production, and determined that these cytokines are detrimental to the host during *B. abortus* infection. It was shown that macrophages and splenocytes exposed to *B. abortus* produce type I IFNs, in agreement with previous studies [35, 144]. Additionally, mice lacking the type I IFN receptor have improved disease outcome upon infection. In vitro examination of splenocytes from wild-type mice and IFNAR−/− mice show that IFNAR−/− splenocytes secrete increased levels of IFN-*γ* and nitric oxide. Thus, type I IFNs induced by *B. abortus* negatively impact the antibacterial response during infection, favoring its own survival [144]. Furthermore, similar to *L. monocytogenes*, type I IFNs are harmful to the host during *B. abortus* infection because in vivo infection of IFNAR−/− mice shows decreased splenocyte apoptosis and decreased bacterial load [87, 144]. Also in agreement with the detrimental effect of type I IFN-related apoptosis, IFNAR−/− BMDMs express less of the proapoptotic gene, *Trail* [144].

During *B. abortus* infection of BMDMs, type I IFN production occurs in a TLR- and TRIF-independent but MyD88- and IRF3-dependent manner. Type I IFNs are also responsible for expression of interferon-inducible resistance proteins (IRGs) during *B. abortus* infection, an effect similarly independent of TLRs but dependent on MyD88 [36]. These TLR-independent observations contradict the original experiments in which IFN-*α* measurement in serum decreased dramatically upon deletion of TLR9 [35]. However, experiments by Huang et al. were performed with heat-killed *B. abortus* rather than the live strain, 2308. It is also known that RAW 264.7 cells, lacking either STING or RNA Pol III and stimulated with *B. abortus*-derived DNA, have diminished type I IFN responses [144]. However, to date, there are no intracytoplasmic nucleic acid sensors upstream of type I IFN production that signal via MyD88 and act together with STING or RNA Pol III. Thus, these pathways must be studied in greater detail to determine the mechanism of type I IFN induction during *B. abortus* infection.

Though there is evidence for both production and impact of type I IFNs during *B. abortus* infection, contradictory experiments using mice on a Balb/c background suggest type I IFN production is dispensable [145]. This group utilized the same strain of *B. abortus* as de Almeida et al. and determined that type I IFN-induced genes were expressed to similar magnitudes in both Balb/c and C57BL/6 mice. Thus, variation in type I IFN induction across mouse or bacterial strain cannot explain the differences in experimental results. Given the contradictory evidence regarding type I IFNs in *B. abortus*, it is clear that greater efforts are required to fully understand these cytokines and their potential during this infection.

### 4.7. *Legionella pneumophila*

*L. pneumophila* is the causative agent of Legionnaire's disease, a form of pneumonia. *L. pneumophila* can infect and replicate within both human epithelial cells and macrophages [146]. Evasion of the innate immune response is due to the type IV secretion system, Icm/Dot, which secretes *L. pneumophila* products from the bacterial vacuole into the host cell cytosol [147, 148]. IFN-*γ* has long been known to restrict *L. pneumophila* replication within macrophages but type I IFNs can also contribute to macrophage resistance [149]. The first documentation of potential pathways leading to production of type I IFNs during *L. pneumophila* infection was conducted in human lung epithelial cells [150]. In these cells, type IV secretion-competent *L. pneumophila* is required for IFN-*β* production and bacterial control. Furthermore, induction of IFN-*β* by *L. pneumophila* or B-DNA occurs downstream of IRF-3 and CARD-containing protein, IPS-1, but does not involve other CARD-containing proteins like RIG-I and MDA5 or the inflammasome protein, ASC [150]. These results are perplexing as no other CARD-containing proteins besides those investigated in this study exist that could explain how DNA is inducing type I IFNs via IPS-1.

In addition to DNA-dependent type I IFN production, RNA-dependent type I IFN production can occur as well. *L. pneumophila* can induce type I IFNs in an RNA-RIG-I-IPS-1-dependent manner [151]. In contrast to the work done by Opitz et al. in a human epithelial cell line, DNA-induced type I IFNs do not require IPS-1, and RNA but not DNA is the primary inducer of type I IFNs in BMDMs [151]. Furthermore, Pol III can transcribe *L. pneumophila* DNA into a RIG-I ligand. This pathway is required for subsequent antibacterial type I IFN induction in murine monocyte/macrophage cell lines [47]. Finally, *L. pneumophila* CDNs are also sufficient for type I IFN production [152]. The latter may be explained by cGAS and STING-dependent signaling as STING-dependent CDN stimulation and type I IFN production have been demonstrated [153]. It is obvious that many different intracellular nucleic acid-detection pathways are activated during *L. pneumophila* infection and results may depend on cell type.

Upon receptor binding and STAT signaling, type I IFNs induce macrophages to differentiate into classically activated inflammatory macrophages, which can produce nitric oxide. This coordinated response is dependent on TBK-1 and IRF3 but occurs independently of STAT1 and STAT2 [154]. The protective effects of type I IFNs during *L. pneumophila* infection are lost in STAT1/2 double knockout macrophages but remain intact when either or both STAT1/2 are expressed [155]. Thus, STAT2 may be compensatory in the absence of STAT1. In this model of type I IFN-induced STAT activation, STAT2 forms a complex with IRF9, which is unique from ISGF3 (STAT1, STAT2, IRF9) [155]. Furthermore, type I IFNs suppress bacterial numbers in macrophage vacuoles in vitro. In vivo, however, protective effects are dependent on both IFN-*γ* and type I IFNs [153]. While evidence in vitro suggests the importance of type I IFNs in restricting bacterial growth in macrophages, they may be dispensable during in vivo *L. pneumophila* infection [151, 156]. These complex results and the multitude of signaling pathways engaged to induce type I IFNs during *L. pneumophila* infection, illustrate the importance of understanding these cytokines during bacterial infection.

### 4.8. *Coxiella burnetii*

*C. burnetii* is an intracellular pathogen of the lung and the cause of Q fever. Permissive subsets of alveolar macrophages and recruited monocytes are believed to be the primary cellular targets of *C. burnetii* infection [157]. In these cells, *C. burnetii* inhabits the macrophage phagolysosome that is extensively modified by bacterial protein [158–160]. Interference with inflammatory proteins like TNF-*α*, IL-6, and IFN-*γ* [161, 162] and production of anti-inflammatory cytokines like IL-10 are hallmarks of infection with virulent *C. burnetii* [163–165]. However, knowledge of the innate immune response during acute Q fever is still lacking. Our group recently determined that, despite the phylogenetic and pathogenic similarities between *L. pneumophila* and *C. burnetii*, pathogen-induced type I IFNs affect the host differently [166]. We determined that during *C. burnetii* infection in mice, type I IFN production negatively impacted the host as displayed by decreased disease in IFNAR−/− mice. Yet the role of type I IFNs was tissue dependent. We compared peripheral to lung delivery of type I IFNs. The results indicated that peripheral delivery of IFN-*α* exacerbated disease, whereas IFN-*α* at the site of infection, in the lung, ameliorated disease. The negative impact of peripheral type I IFNs on the host was hypothesized to be due to the ability of type I IFNs to decrease inflammatory cytokine expression [167]. At this time, the *C. burnetii*-derived ligands and pathways that are responsible for type I IFN induction are just beginning to be understood. *C. burnetii* encodes all but three proteins involved in the type IVB secretion system of *L. pneumophila*; so, it is plausible that *C. burnetii* is sensed and induces type I IFNs in a manner similar to that of *L. pneumophila* [168]. Additionally, *C. burnetii*-stimulated pDCs make IFN-*α* and have upregulated expression of genes upstream of type I IFN production like *TLR7/9*, *IRF7*, *MyD88*, *RIG*-*I*, *NOD1*, and *NOD2* [169]. Thus, *C. burnetii* may induce production of type I IFNs in many ways, though pathways likely differ between cell types. Our results provide an excellent example of the need to assess the impact of type I IFNs on different tissue sites during bacterial infection.

## 5. Concluding Remarks

**Figure 1:**
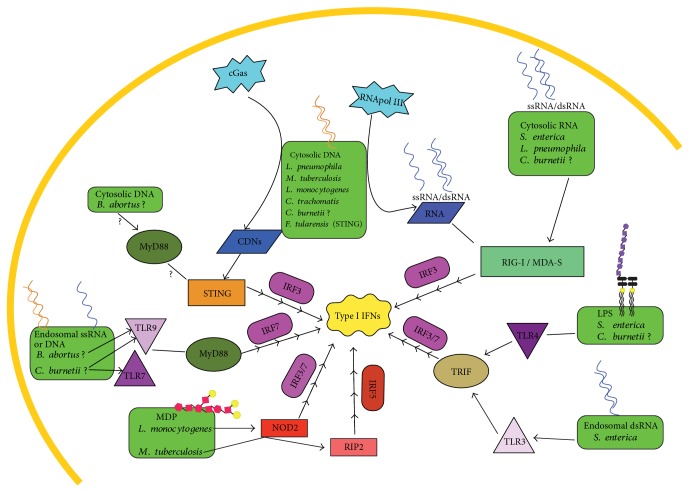
Induction of type I IFNs by intracellular bacteria. Type I IFNs can be induced via cytosolic nucleic acid sensors RIG-I, MDA5, RNA Pol III, cGAS, and STING or nucleic acid-sensing TLR3, 7 or 9. Bacterial products like LPS and MDP can signal the production of type I IFNs via TLR4 or NOD2/RIP2, respectively.

Type I IFNs are potent immunomodulatory signaling molecules and have the capacity to diversely affect immune responses. Our knowledge of these signaling molecules has expanded greatly since their discovery, and now, we know they are induced during intracellular bacterial infections and have a multitude of effects (Figure 1, Table 1). The evolutionary importance of type I IFN production in this context is clear, as many intracellular bacteria have devised methods of co-opting type I IFN production for their benefit. Enhancing means of cell death and dampening inflammatory responses serve as excellent examples of this, but these effects could also benefit the host. The downstream effects of type I IFNs depend on the route of infection, which PRR signaling pathways are engaged, and the presence of virulence factors that allow for intracellular detection of bacterial components. In each of the intracellular bacteria reviewed here, there is no clear-cut evidence as to the effects of type I IFNs being only beneficial or only detrimental to the host. This is not surprising because minute differences in the magnitude of type I IFN protein expression and expression of IFNAR on cell surfaces can tip the balance and define the impact of type I IFNs. Thus, the web of type I IFN signaling is more complex than previously thought. When type I IFNs are part of the immune response in a given setting, everything from host genetics and health history to current disease state must be evaluated. It has been shown in mice that influenza A-induced type I IFNs increase mycobacterial growth and disease in coinfected animals [170]. Thus, secondary infections during *M. tuberculosis* with pathogens that may induce type I IFNs could have a drastic impact on antimycobacterial host responses. Furthermore, treatment for *C. burnetii* infection is an aggressive long term antibiotic regimen, and considering intratracheal delivery of type I IFNs is beneficial in a mouse model of disease, perhaps aerosolized type I IFNs may be an effective alternative to antibiotics [167, 171]. Situations like this highlight the need for more detailed studies to better understand how, when, and why type I IFNs may benefit the host or pathogen. This may open new avenues of treatment options beyond antibiotics to combat intracellular bacterial infections.

## Figures and Tables

**Table 1:** Mechanisms of type I IFN induction by intracellular bacteria and downstream consequences of induction. Type I IFNs are induced via various signaling pathways depending on the bacterial ligand and host cell type. Repercussions of type I IFN production differ between intracellular bacteria, cell type infected, and model/route of infection among other factors.

Bacteria	Gram stain	Induction of type I IFN	Role of type I IFN	Effects on host	References
*C. trachomatis/muridarum*	−	cGAS generates cGAMP from *Chlamydial* DNA which activates STING/IRF3	(1) Beneficial	(1) Depletes intracellular iron and regulates IDO which can alter tryptophan availability to bacteria; IFN-*α* together with IFN-*γ* or TNF-*α* has synergistic antichlamydial effect	[44, 71–73]
			(2) Detrimental	(2) Decreased chlamydia-specific T cells and decreased CXCL9-induced T cell recruitment prevent bacterial clearance	

*L. monocytogenes*	+	DNA- or CDN-mediated signaling via IFI16 and STING, RNA signaling via RIG-I or MDA5, RNA Pol III transcription of DNA to RNA, NOD2 detection of MDP in conjunction with cytosolic detection of DNA	(1) Beneficial	(1) Decreases inflammatory pathology in liver and induces rapid protective cytokine response after intragastric infection	[43, 49, 74, 79–84, 87–89, 91–93, 95]
			(2) Detrimental	(2) Increases expression of apoptotic genes and increases apoptosis of lymphocytes and macrophages needed for bacterial clearance, decreases production of antibacterial IL-12p70, and decreases TNF-*α* secretion from macrophages; dampened macrophage responsiveness to IFN-*γ* makes these cells more permissive to infection/replication, decreases numbers of IL-17A-secreting *γδ* T cells thereby decreasing neutrophil recruitment and bacterial control in the spleen, increases cell-to-cell spread of bacteria, and inhibits CD8 T cell priming	

*M. tuberculosis*	NA	cGAS-dependent generation of CDNs which activate STING, NOD2 detects *Mycobacterial* MDP leading to activation of RIP2 and IRF5	(1) Beneficial	(1) Hypervirulent strain promotes alternative macrophage activation in the absence of IFN-*γ* that controls bacterial replication	[20, 41, 42, 98, 105–107]
			(2) Detrimental	(2) Dampens antibacterial IL-1*β* production, IL-10 downstream of type I IFNs reduces antibacterial activity of IFN-*γ*; causes fatal inflammation due to increased chemokine production, neutrophil recruitment to the lung, and alveolar macrophage death	

*S. enterica* serovar Typimurium	−	TRIF-dependent TLR3 and TLR4 signaling likely via nucleic acids and LPS, respectively, RIG-I detection of RNA	(1) Beneficial	(1) Important for antibacterial macrophage responses	[111–114, 118, 120–122, 124]
			(2) Detrimental	(2) Increased caspase-11-mediated macrophage death allowing bacterial release which is exacerbated in the absence of caspase-1, a protein required for neutrophilic control of infection, increases RIP1/RIP3-mediated macrophage death; suppresses IL-1 cytokine and neutrophil chemoattractant transcripts in macrophages which decreases bacterial control; influenza-induced type I IFNs negatively impact gut microbiota and decrease innate responses in the gut, increasing susceptibility to *S. enterica*	

*F. tularensis*	−	cGAS, IFI204, STING, and IRF3-dependent	(1) Beneficial	(1) Type I IFN-induced GBPs activate AIM2 inflammasome leading to macrophage pyroptosis and removal of replicative niche	[91, 126, 135–139]
			(2) Detrimental	(2) Decreased number of IL-17A-secreting *γδ* T cells which are important for neutrophil recruitment to the spleen and bacterial control	

*B. abortus*	−	RNA Pol III and/or STING are required for type I IFN induction in a MyD88-dependent but TRIF- and TLR-independent manner	(1) Detrimental	(1) Alters DC maturation which dampens DC cytokine production; increases bacterial load due to decreases in IFN-*γ* and NO production; increases splenocyte apoptosis and *Trail* expression in macrophages	[36, 141, 144]

*L. pneumophila*	−	RIG-I detection of RNA and subsequent signaling through IPS-1 and IRF3, RNA Pol III-dependent transcription of DNA to RNA then signaling via RIG-I and IPS-1, CDN detection via STING	(1) Beneficial	(1) Controls bacterial replication within host cell vacuoles; promotes inflammatory macrophage polarization and bacterial clearance	[47, 82, 150–154]

*C. burnetii*	−	NOD1/2, RIG-I, and/or TLR7/9-mediated production of IFN- *α* in pDCs, involves IRF7	(1) Beneficial	(1) Administration to lung was protective—mechanism unknown at this time	[167, 169]
			(2) Detrimental	(2) Lack of IFNAR benefited host; i.p. delivery of IFN-*α* was harmful to host perhaps due to suppression of necessary inflammatory cytokines	

## Abbreviations

AIM2: Absent in melanoma 2
BMDM: Bone marrow-derived macrophages
CDN(s): Cyclic dinucleotide
cGAMP: Cyclic GMP-AMP
cGAS: Cyclic GMP-AMP synthase
CLR(s): C-type lectin receptor
GBP(s): Guanylate binding protein
IFN: Interferon
IFNAR: Type I IFN receptor
MDP: Muramyl dipeptide
NLR(s): NOD-like receptor
PAMPs: Pathogen-associated molecular patterns
PRRs: Pattern recognition receptors
RIP: Receptor interacting protein/receptor interacting protein kinase
RLR(s): RIG-I-like receptor
SesT: *Salmonella enterica* serovar Typhimurium
STING: Stimulator of interferon genes
TLR(s): Toll-like receptor.
